# Efficacy and tolerability of lamivudine in hepatitis B infected renal transplant recipients: A single center study

**DOI:** 10.4103/0971-4065.57104

**Published:** 2009-07

**Authors:** S. K. Agarwal, S. C. Tiwari

**Affiliations:** Department of Nephrology, All India Institute of Medical Sciences, New Delhi, India

**Keywords:** Efficacy, India, lamivudine, renal transplant, tolerability

## Abstract

Hepatitis B virus (HBV) infection in patients on hemodialysis and renal transplantation (RT) usually has an unfavorable course. Lamivudine is a synthetic nucleoside analog with a potent action on HBV replication. There is limited data on lamivudine in renal transplant patients with HBV infection and no published report from India. Present study reports on lamivudine therapy in these patients. Patients with HBV infection taken for RT were included. Hepatitis B surface antigen (HBsAg), hepatitis B envelope antigen (HBeAg), HBV-DNA, and liver biopsy before RT were done in all patients. Lamivudine was given in the dose of 50 mg daily during the dialysis and 100 mg daily following successful transplant. Response was evaluated at one year. Of the 739 adult RTs during study period, 35 (4.7%) had HBV infection. Mean age of patients was 30.7 ± 9.8 (16–55 years) and 88.5% were males. Four (11.4%) patients had HCV coinfection. HCV was not treated in any patient. All patients were HBsAg and HBV-DNA positive, while 27 (77%) were HBeAg positive. Mean ALT was 77.8 ± 90 IU/dl; 11 (31.4%) patients had normal ALT. Mean liver biopsy grade was 5.2 ± 1.5 (3–9) and stage was 0.7 ± 0.6 (0–2). At one year following transplantation, ALT was normal in 27 (77%) cases, HBV-DNA undetectable in 16 (45.7%), HBeAg and HBsAg seroconversion in 8 (22.8%), and 3 (8.6%) cases, respectively. All patients tolerated the drug without any significant side effects. Treatment with lamivudine in dialysis and renal transplant patients is well tolerated and safe with efficacy comparable to patients with normal renal function.

## Introduction

Hepatitis B virus (HBV) infection in patients with endstage kidney disease (ESKD) subjected to renal replacement therapy (RRT), hemodialysis, and/or renal transplantation (RT) usually has an unfavorable course with a tendency toward chronicity leading to increased morbidity and mortality.[1] Though its incidence in patients with ESKD has decreased significantly due to preventive measures, like vaccination and practices of universal precaution and isolation during dialysis, this infection still continues to occur during RRT in many units in the world including India. The frequency of HBV seropositivity in RRT in India varies from 4–44%.[2–4] Higher percentage is seen only in very few units. In most of the larger units it is less than 5%. In absence of good maintenance hemodialysis (MHD) program in the country, these patients regularly get transplant unless they have significant histological fibrosis or clinically advanced liver disease. We had reported earlier that chronic liver disease (CLD) due to hepatitis was second commonest cause of death in renal transplant patients in second decade.[5] The impact of RT and treatment with immunosuppressive drugs on patients with HBV is not very clear. Immunosuppressive therapy does modify the natural history of the hepatic disease due to the increase in viral replication,[6] though the real impact on patient outcome in HBV-infected patients usually does not become apparent in initial few years of RT[7,8] after which liver failure is responsible for death in good number of cases.[5] Interferon used for the treatment of HBV infection has been shown to be effective in large number of cases, but in renal transplant patients it is contraindicated as it can induce rejection episodes.[9]

Lamivudine is a synthetic nucleoside analog with a potent action on HBV replication, thus decreasing the progression of liver disease.[10] Unlike interferon, lamivudine does not have an immunomodulatory effect and therefore can be safely used in renal transplant patients. There are some studies in literature on the role of lamivudine in these patients;[11–29] however, there is no report from India on efficacy and tolerability of lamivudine in renal transplant patients with HBV infection. The aim of the present study was to report the effectiveness and safety of lamivudine therapy in HBV-positive RT patients.

## Materials and Methods

In this retrospective study, all patients between January 1998 and March 2007 with HBV infection taken on MHD program followed by RT at our institute were evaluated. We excluded pediatric patients (all below 18 years of age) as the pediatric department before and after RT followed them. In addition to routine laboratory work-up, they were investigated for serum bilirubin, alanine aminotransferase (ALT), aspartate aminotransferase (AST), alkaline phosphatase, and prothrombin time using standard laboratory methods. Hepatitis B surface antigen (HBsAg) and hepatitis B envelop antigen (HBeAg) were done using ELISA-based test kit (J Mitra and Co Ltd, India). Qualitative HBV-DNA was done using reagent from QIAGEN Hamberg and sensitivity of test was 20 IU/ml or 120 genomic copies/ml. All patients were subjected to percutaneous liver biopsy before RT, and grading and staging were assessed using the international liver study HAI scoring system. Before the liver biopsy, patients were either subjected to ultrasound abdomen or upper GI endoscopy to assess portal hypertension. After the liver biopsy RT was not done only in a situation, when the biopsy showed histological nodule formation as evidence of cirrhosis. Lamivudine was started orally in the dose of 50 mg daily during the dialysis period as patients with renal failure require reduction by 50% of normal dose and lamivudine is not dialyzable significantly during dialysis, and the dose was increased to 100 mg daily once the renal function returned to normal following transplant. A short stint of dialysis and lamivudine course was followed by the transplant. This is because most of the patients received few dialysis in our hospital, as waiting period for renal transplant was approximately three months with the noncadaver transplant donors being related to patients. So, all the investigations were practically done just before renal transplant. Thus, response to lamivudine during the dialysis period was not evaluated in view of the short time spent by the patients on dialysis in our unit before their transplantation. Lamivudine was continued for more than one year, but we herewith report results of drug treatment at the end of one year. We used triple immunosuppression consisting of calcineurin inhibitor (cyclosporin or tacrolimus), steroid, and azathioprine or mycophenolate mofetil. Details of immunosuppressive protocol have been published earlier.[30] Following transplant, patients were followed twice weekly for first 2–3 months, weekly for next three months, every fortnight for next three months, and then at 3–4 week intervals. At each visit, in addition to clinical examination, hemoglobin, total and differential leukocyte counts, platelets, blood urea, serum creatinine, and serum sodium and potassium were done using standard laboratory methods. Liver function tests consisting of serum bilirubin, AST, ALT, and alkaline phosphatase were done at three months, six months, and at one year. HBsAg, HbeAg, anti-HBe, and qualitative HBV-DNA were repeated at the end of one year. Acute rejection within one year, patient and graft survival was also evaluated at the end of one year, though this was not the main aim of the study. All results were expressed as mean ± SE. Comparison of values obtained before and during lamivudine therapy was done using nonparametric tests. A *P* value of ≤0.05 was considered to be statistically significant.

## Results

From January 1998 till March 2007, 769 renal transplants were performed at our center. Of these, 30 patients were pediatric transplants and were excluded as pediatric team managed these patients separately. Of the remaining 739 adult patients, we had 35 (4.7%) patients with HBV infection. Mean age of patients was 30.7 ± 9.8 years (16–55 years), and 88.5% were males. Basic disease causing ESKD was chronic glomerulonephritis in 22.8%, polycystic kidney disease and vesicoureteric reflux in 5.7% each, diabetic nephropathy in 1 case, and unknown in rest of the patients. Mean number of hemodialysis given was 76.2 ± 39.4 (8–175), and mean number of blood transfusions was 9.4 ± 8.2 (0–60). Four (11.4%) patients had HCV coinfection. None of the patients was treated for HCV infection due to cost of therapy. Mean duration of hemodialysis during which patients received lamivudine before RT was 9.5 ± 1.6 weeks (3–19 weeks). Four (11.4%) patients were also on antitubercular drugs at the time of transplant. In antitubercular drug therapy in renal transplant, we do not use rifampicin. In place of rifampicin we use ofloxacilin as a part of regular therapy. All patients received transplants from living related donors – parents: 45.7%, siblings: 28.5%, spouse: 22.9%, and unrelated: 3%. Seventy one point five percent donors were females. None of the donors had HBV infection as we do not accept HBV-infected living donor as a policy in the department. Of the 35 patients, 31 received cyclosporin and 4 tacrolimus, while 28 patients received azathioprine and 7 patients mycophenolate mofetil. None of the patients received induction immunosuppression. All patients were HBsAg and HBV-DNA positive but only 27 (77%) were HBeAg positive. Mean ALT was 77.8 ± 90 IU/dl (13–570), and 11 (31.4%) patients had ALT level below the upper limit of normal for our lab (≤50 IU). Mean liver biopsy grade was 5.2 ± 1.5 (3–9) and stage was 0.7 ± 0.6 (0–2). During follow-up period, eight (22.8%) patients had at least one acute rejection. Of these eight patients, five patients had acute rejection within three months following transplant and two patients had second acute rejection. However, there was no difference in the response rate of lamivudine for HBV viral infection in patients who were given antirejection as compared to patients who never got any antirejection therapy. At one year following transplantation, ALT was normal in 27 (77%) cases, HBV-DNA was undetectable in 16 (45.7%), HBeAg seroconversion and development of anti-HBe was seen in eight (22.8%) cases, and HBsAg seroconversion was seen in three (8.6%) cases. These results as well as comparative results of other published studies have been shown in Table 1. All the patients tolerated the drug very well without any significant side effects attributable to drug itself. With a mean follow-up of 47.8 ± 35.6 (1–105) months, seven patients died. Three patients had bacterial pneumonia, three septicemia and one cryptococcus pneumonia. Patient and graft survival was 85% at the end of one year in these patients as compared to 89% in HBV-negative patients. As the second transplant is very uncommon in our set-up, graft survival is almost similar to patient's survival and patients usually lost to follow-up once the graft fails. We as such did not compare the results of HBV-infected patients with uninfected patients due to gross difference in the number of patients, HBV-negative patients being in large number as compared to HBV-positive patients being only 35.

**Table 1:** Comparison of studies on lamivudine in HBV-positive patients in dialysis and renal transplant

Year	Author	Country	Ref No.	No	Age (Yrs)	Males (%)	LAM Dose	Eag +ve (%)	DNA +ve (%)	HALT (%)	CIR (%)	DT Mon	ALTN (%)	SagE (%)	PCRN (%)	EagE (%)	LR (%)
2008	Present study	India	NA	35	30	88	50–100	77	100	68.6	0	12	77	8.6	54.3	22.8	NA
2006	Filik	Turkey	28	15	NA	NA	100	NA	13	NA	NA	6	100	NA	NA	NA	NA
2005	Lapinski	Poland	27	7	27–58	57	100	NA	100	NA	NA	12	100	NA	14	43	29
2004	Kamar	France	26	18	46	77	100	22	100	77	28	36	72	NA	55	25	NA
2002	Santosh	Brazil	25	6	37	66	150	100	100	NA	0	24	NA	0	100	NA	33
2002	Chan	Hong Kong	24	26	47	81	100	54	100	54	0	32	100	0	100	21	42
2002	Park	Korea	23	10	30	90	100–150	50	100	100	0	35	80	0	70	20	10
2001	Lee	Taiwan	22	13	34	85	100–150	61	100	100	NA	12	NA	8	77	37	25
2001	Han	Korea	20	6	35	83	100	50	100	100	17	12	100	0	100	67	50
2001	Mosconi	Italy	21	4	33	75	50–100	NA	100	100	NA	23	NA	0	100	NA	0
2000	Kletzmayr	Taiwan	19	19	49	79	100	63	84	16	NA	12	100	0	93	17	25
2000	Tsai	Austria	18	11	40	54	100	NA	73	100	NA	NA	NA	0	87	0	0
2000	Lewandowska	Poland	15	28	46	75	75–150	93	36	100	14	4	61	0	100	8	0
2000	Antoine	France	17	12	50	75	NA	100	100	NA	NA	9	NA	0	75	67	0
2000	Mouquet	France	16	15	38	NA	50–100	27	100	100	40	6	53	0	87	NA	27
2000	Fontaine	France	14	26	47	65	100	50	100	NA	31	16	NA	0	100	46	31
1999	Goffin	Belgium	13	4	52	100	100	25	100	100	33	12	100	0	100	0	25
1998	Jung	Korea	12	6	35	100	100	50	100	100	NA	8	100	0	100	33	0
1997	Rostaing	France	11	6	49	83	100	17	100	83	33	6	80	0	100	0	0

LAM: Lamivudine, Eag: HbeAg, HALT: High ALT, CIR: Cirrhosis on histology, DT Mon: Duration of treatment in months, ALTN: ALT normalization, SagE: HbsAg elimination, PCRN-HBV:PCR normalization, EagE: HbeAg elimination and development of anti-HBe, LR: Lamivudine resistance, NA: Not available

## Discussion

Treatment of chronic hepatitis B patients undergoing hemodialysis followed by RT is an important issue at places where this infection is still seen. Interferon, one of the standard therapies of HBV infection, is contraindicated in renal transplant patients and its tolerability is poor in dialysis patients. Thus, lamivudine has been commonly used drug to treat HBV infection in these patients. Though entecavir is a promising new drug for HBV infection, which is said to be better than lamivudine for naive patients, its use in renal transplant patients is yet to be studied. Till then lamivudine will continue to be a commonly used drug in these patients. Our experience showed that although HBV-PCR clearance is possible in nearly 50% patients, HBeAg and HBsAg seroconversion is seen in very few patients. Enzyme normalization was seen in 77% patients. However, enzyme normalization is difficult to interpret in these patients, as many of such patients being immunosuppressed may not show enzyme elevation in spite of having disease activity on histology. Practically in these patients, probably we have to depend on virological response for assessing therapy rather than response to ALT normalization. In terms of sample size, this study by far had the largest number of renal transplant patient series in whom the role of lamivudine was assessed. There have been other studies on the role of lamivudine in renal transplant patients. A comparison of majority of these studies is shown in Table 1. In most of the studies, a majority of patients were males and mean age was in third and fourth decades as in our patients. HBeAg positivity was variable ranging from 17–100% patients while in our study it was 77%. In five studies[14,17,25,27–28] liver enzyme status at the time of starting lamivudine was not available, while in the remaining studies, ALT was elevated in most of the patients. Liver biopsy findings were not available in eight studies.[12,17–19,21,22,27,28] In studies where biopsy findings were available, cirrhosis was seen in nearly one-third patients, while in our study, none of the patients had cirrhosis on histology. Patients on maintenance hemodialysis are tested for HBsAg on monthly basis as a policy in all the units. Thus, in these patients, it is possible to time the onset of HBV infection as against general population where timing of infection may be difficult to assess. Further, waiting period for renal transplant in our unit is few months only. Thus, our patients had short duration of HBV infection, with mean timing on dialysis being six-and-a-half months only, which is unlikely to produce cirrhosis. Duration of treatment in other studies with lamivudine was variable from 4–36 months. We continued therapy beyond 12 months but result analysis was done at the end of one year. We did not evaluate lamivudine resistance.

Enzyme normalization status was not available in six studies,[14,17,18,21,22,25] while in rest of studies it was seen in 53–100% patients. Hepatocytes damage in the course of chronic hepatitis B is mainly immune mediated, in addition to a less significant role of direct HBV hepatotoxicity. Immune suppression in renal transplant patients as well as patients on dialysis could diminish specific anti-HBV responses, which could alter immune-mediated cytopathic effects. This could result in the decrease of hepatic inflammation and low ALT activity. Thus, in patients on dialysis and RT, the cut-off value of ALT is controversial and is definitely lower than patients with normal renal function. Therefore, unless cut-off value in these patients is standardized to local population, it will be difficult to interpret normalization of enzymes in different studies. In treatment of patients with chronic HBV infection probably best can be initiated depending upon quantitative viral load and/or liver biopsy, and enzymes can be of less value for deciding therapy. Though HBV-PCR clearance has been 14–100%, in most of the studies it has been in more than two-thirds of cases. In our patients, virus could be cleared in 54.3% cases. Though we could not study lamivudine resistance, some studies in past have evaluated lamivudine resistance in similar patient population and found lamivudine resistance in 10–50% patients with variable duration of therapy. We did not observe significant side effects of lamivudine treatment in patients either on MHD period or following RT. Park *et al*,[23] presented similar observations concerning the safety and tolerability of lamivudine treatment in renal transplant patients.

Twenty two point eight percent patients had acute rejection and it was similar to overall incidence of acute rejection in our patients, though the group has not been compared statistically with HBV-negative patients due to gross disparity in the number of cases. Graft and patient survival was also similar to other patients. With only one year of evaluation, we do not expect any change in patients and graft survival in these patients due to HBV infection.

With scientific advancement, therapy of HBV infection is changing with time. As against interferon for HBV that is a time-bound therapy, duration of therapy with synthetic nucleoside analog is not defined both in nonrenal and renal transplant patients. With the availability of newer drugs like entecavir and tenofovir, which have become first line of therapy in naive HBV-infected patients, we are likely to use these drugs in renal transplant patients also.[31] However, in transplant patients it is time that will tell the efficacy and tolerability of these drugs; as in most of the clinical trials on these drugs, transplant patients have been excluded and currently there is paucity of studies with these drugs in renal transplant patients.

We have two limitations in this study. Firstly, this is a retrospective study. Secondly, in HBV-infected patients being treated, quantitative decrease in DNA titer and resistance to lamivudine are important issues, which were not studied in our patients.

In conclusion, treatment with lamivudine in dialysis patients as well as in renal transplant patients is well tolerated and safe. Effectiveness of lamivudine therapy in these patients is comparable to patients with normal renal function. Till the other drugs against HBV infections are studied in these patients, in view of low cost, reasonable response, and good tolerability, lamivudine may be used in these patients.
